# Effect of Orthodontic Tooth Movement on Sclerostin Expression in Alveolar Bone Matrix: A Systematic Review of Studies on Animal Models

**DOI:** 10.3390/dj13110513

**Published:** 2025-11-04

**Authors:** Meredith L. Rogers, Paul Emile Rossouw, Fawad Javed

**Affiliations:** Department of Orthodontics and Dentofacial Orthopedics, Eastman Institute for Oral Health, University of Rochester, Rochester, NY 14620, USA; meredith_rogers@urmc.rochester.edu (M.L.R.); emile_rossouw@urmc.rochester.edu (P.E.R.)

**Keywords:** sclerostin, SOST, orthodontic tooth movement, tooth movement techniques

## Abstract

**Background/Objectives**: Sclerostin is a glycoprotein produced by osteocytes that regulates osseous remodeling, particularly in the context of orthodontic tooth movement. The purpose of the current systematic review is to assess the effect of orthodontic tooth movement (OTM) on sclerostin expression (SE) in the alveolar bone matrix (ABM). **Methods**: Indexed databases including PubMed, Embase and Web of Science were searched without time and language restrictions up to and including March 2025. **Results**: Seven studies performed on 8- to 12-week-old male rodents were included. The magnitude of orthodontic forces ranged from 10–120 g. Distalization and mesialization of the maxillary first molar were performed in one and six studies, respectively. In two studies, SE was increased on the compression and tension sides during OTM. In one study, SE is increased and decreased on the compression and tension sides, respectively; and another reported no difference in SE on the compression and tension sites during OTM. Two studies did not report data on SE on the control-sites (sites unexposed to OTM). Sample-size estimation was not performed in any of the included studies. All studies had a high risk of bias (RoB) and low certainty of evidence (CoE). **Conclusions**: Sclerostin may play a regulatory role in ABM during OTM. However, current evidence is limited by methodological inconsistencies, high RoB, and low CoE. Well-designed, power-adjusted studies using standardized protocols are required to establish reproducible findings and assess the translational potential of SE in orthodontics.

## 1. Introduction

Orthodontic tooth movement (OTM) is a regulated biological process that occurs when mechanical forces are exerted on teeth, inducing remodeling of the surrounding tissues [1]. This remodeling involves osseous resorption and formation on the pressure and tension side of the alveolar bone, respectively, leading to the gradual movement of teeth [2,3]. The orchestration of these processes is complex and involves numerous cellular events, including the recruitment of osteoclasts, osteoblasts, and osteocytes. Moreover, intercellular signals that stimulate cellular processes needed for OTM encompass receptor activation of nuclear factor-κB ligand (RANKL), tumor necrosis factor-α, transforming growth factor-beta, and bone morphogenetic proteins [4,5,6]. However, other factors that influence the rate and extent of OTM include the magnitude of force applied, patients’ age, and individual variability in bone density and metabolic activity [7,8,9,10].

Sclerostin is a glycoprotein produced by osteocytes that has emerged as a key regulator of osseous remodeling, particularly in the context of OTM [11,12,13,14,15,16,17]. Sclerostin functions as an inhibitor of the Wingless/Integrated signaling pathway/beta-catenin pathway, which is crucial for bone formation and remodeling [18]. In orthodontics, the sclerostin gene/SOST is studied for its implications in mechanotransduction, alveolar bone turnover and potential therapies for modulation of bone remodeling [19,20]. Moreover, osteocytes act as “mechanotransductors” by responding to mechanical stimuli through a series of biological signaling cascades by modulating their release of sclerostin [13,21,22]. Through this mechanism, OTM facilitates controlled bone remodeling, thereby enabling effective tooth movement. In an experimental study on rats, Lu et al. [14] assessed the in vivo effects of local sclerostin injections on OTM. In this study [14], orthodontic mesialization of the bilateral maxillary first molars was performed with a local injection of sclerostin protein on the test-side, and the same volume of normal saline was injected on the control side. The two-week follow-up results showed that OTM was significantly greater in the test sites than in the control sites [14]. The study concluded that local sclerostin injection in the compression side of alveolar bone increases OTM by promoting osteoclastogenesis [14]. Likewise, a histologic study on Wistar rats investigated the expression of sclerostin during experimental OTM and its effect on periodontal tissue remodeling [15]. The results showed a higher expression of sclerostin and the number of osteoclasts on the compression side than on the tension side during experimental OTM [15]. Similar results were reported by Odagaki et al. [23] and Shu et al. [16]. In this context, it is critical to comprehend the role of sclerostin and its therapeutic implications in relation to OTM; however, a systematic review of studies assessing the influence of OTM on sclerostin is yet to be documented.

The purpose of the present systematic review preclinical studies that assessed the effect of OTM on sclerostin expression (SE) in the alveolar bone matrix (ABM).

## 2. Materials and Methods

### 2.1. Protocol and Registration

The present study was conducted in accordance with the guidelines established by the Preferred Reporting Items for Systematic Reviews and Meta-Analyses (PRISMA) [24]. The protocol was registered with the International Prospective Register of Systematic Reviews (PROSPERO) before the commencement of the present study (PROSPERO Registration #CRD42025586571).

### 2.2. Focused Question and PICO

The focused question “Does OTM influence SE in the ABM?” was framed using the Population, Intervention, Comparison, Outcome (PICO) framework as follows: P: Animal models subjected to OTM; I: application of orthodontic forces to teeth/OTM (test-group); C: no OTM (control-group); and O: SE in the ABM.

### 2.3. Eligibility Criteria

Studies with the following characteristics were considered eligible for inclusion: (a) original studies that assessed the expression of sclerostin in the ABM in relation to OTM; and (b) studies with a control-group (no OTM). In silico and ex vivo studies, case-reports, case-series, commentaries, reviews and perspectives were excluded.

### 2.4. Literature Search Protocol

A comprehensive search of electronic databases (PubMed, Web of Science, and Embase) was conducted without time and language restrictions up to and including March 2025. A comprehensive electronic search was conducted using the following terms combined with Boolean operators: “sclerostin” OR “SOST” AND “orthodontic tooth movement” OR “tooth movement techniques.” Manual searches were conducted by screening the references of relevant original and review articles to identify any study/studies that remained unidentified during the search of electronic databases. The literature search was independently performed by two authors (MLR and FJ). Disagreements were resolved via discussion and consultation with a third author (PER).

### 2.5. Data Extraction

The following information was extracted from each included study: (a) author et al.; (b) year of publication; (c) study participants/subjects (n); (d) age of participants/subjects; (e) gender of subjects/participants; (f) weight of subjects; (g) study groups (test- and control-groups); (h) site of OTM; (i) mode of OTM; (j) force of OTM; (k) duration of OTM; (l) results; (m) conclusion; and (n) sample-size estimation (SSE).

### 2.6. Risk of Bias Assessment

The risk of bias (RoB) was assessed using the Systematic Review Centre for Laboratory animal Experimentation (SYRCLE) tool [25]. Each study was assessed using the following ten domains: (a) sequence generation; (b) baseline characteristics; (c) allocation concealment; (d) random housing; (e) blinding of investigators; (f) random outcome assessment (AC); (g) blinding of outcome assessor; (h) incomplete outcome data; (i) selective outcome reporting; and (j) other sources of bias. Each of the above domains was rated as “low risk”, “high risk”, “critical risk” or “unclear risk” based on the information provided in the study. The RoB within each study was individually appraised by two authors (MLR and FJ). Disagreements were settled via discussion and consultation with a third author (PER).

### 2.7. Grading of Recommendations, Assessment, Development, and Evaluations

The Grading of Recommendations, Assessment, Development, and Evaluations (GRADE) analysis was performed to assess the quality of evidence and the strength of recommendations [26]. Five key domains were used for GRADE analysis—(a) RoB; (b) inconsistency; (c) indirectness; (d) imprecision; and (e) publication bias. The overall quality of the evidence for each outcome was classified as “high”, “moderate”, “low” or “very low”. The GRADE analysis was individually performed by two authors (MLR and FJ). Disagreements were reconciled via discussion and consultation with a third author (PER).

## 3. Results

### 3.1. Study Selection

**Figure 1:**
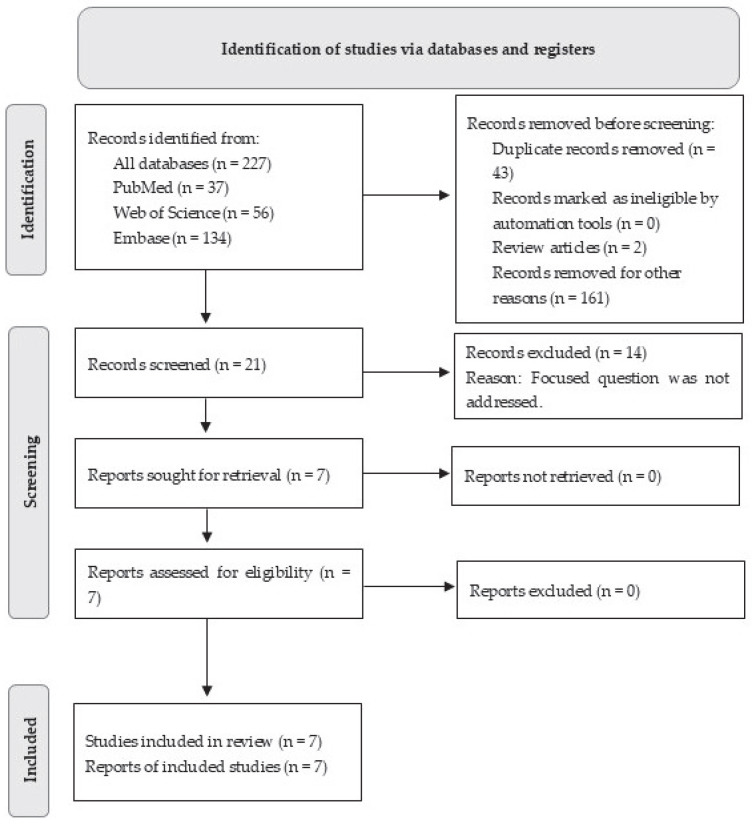
PRISMA flow diagram for the review process.

A total of 227 records were identified through database searches across multiple searches (Supplementary Table S1). After removing four duplicate records and two review articles, 182 studies were further screened. Of these, 175 were marked ineligible as they did not address the focused research question. Seven studies [12,15,16,17,20,22,23] performed on animal models were included and processed for data extraction (Figure 1).

### 3.2. Study Characteristics

All studies [12,15,16,17,20,22,23] were performed with male rodents with ages ranging between 8–12 weeks. Five studies [12,15,16,17,22] reported the population size of the studies, which included 4–35 rats or mice. Four studies [12,15,16,20] reported the weight of rats, which ranged from 180–220 g (Table 1). Prior SSE was performed in none of the studies [12,15,16,17,20,22,23].

### 3.3. Study Characteristics Relating to Orthodontic Tooth Movement

The characteristics of the experimental and control groups varied among the included studies [12,15,16,17,20,22,23]. In four studies [15,17,20,22], animals in the test and control groups underwent experimentally induced OTM and no treatment, respectively. In the studies by Odagaki et al. [23] and Shu et al. [16], SE was assessed between 0 and 10 days and 1 and 21 days of OTM, respectively. In the study by Nam et al. [12], SE was assessed on days 1, 2 and 6 of biophysical force tooth movement. The magnitude of orthodontic forces applied was reported in six studies [12,15,16,17,22,23], which ranged from 10 to 120 g. Distalization and mesialization of the maxillary first molar were performed in one [20] and six [12,15,16,17,22,23] studies, respectively. Two studies [12,20] did not report the duration after which animals were euthanized, and in the remaining studies [15,16,17,22,23], euthanasia was performed after 0 to 14 days. The SE was assessed via immunofluorescence in three studies [12,17,23] and via immunohistochemistry in three studies [15,16,20]. In one study [22], SE was evaluated using an enzyme-linked immunosorbent assay (ELISA) (Table 2).

### 3.4. Sclerostin Expression

#### 3.4.1. Sclerostin Expression on Test-Sides (Sites Exposed to OTM)

In two studies [12,15], SE was increased on the compression and tension sides during OTM. In the study by Yiwen et al. [15], a statistically significant increase in SE was observed at the fifth day of OTM compared with baseline. In two studies [16,23], SE increased on the compression side compared with the tension side during OTM. Results by Shu et al. [16] showed that SE is increased and decreased on the compression and tension sides, respectively, on the first day of OTM. This study [16] also showed that SE remains elevated for one week on compression sides after application of orthodontic forces and the decreases gradually. Nishiyama et al. [17] showed that there is no difference in SE on the compression and control sides and is decreased on the tension side. One study [12,20] reported that SE is decreased on the compression and tension sides. In the study by Yan et al. [22], the concentration of sclerostin was significantly higher in sites exposed to OTM (22.85 pg/mL) compared to the control sites (14.18 pg/mL) (*p* < 0.01). This study [22] did not report any differences in SE among tension and compression sites, specifically in the OTM group. Most of the studies [12,15,16,17,20,23] did not report numerical data in regard to SE (Table 3).

#### 3.4.2. Sclerostin Expression on Control-Sides (Sites Unexposed to OTM)

Two studies [20,23] report no data on SE on control sides. According to Nam et al. [12] and Yiwen et al. [15], the control sites exhibit minimal and undetectable SE, respectively. One study [16] reported that there is no difference in SE on the mesial and distal sites, whereas results by Nishiyama et al. [17] showed higher SE on the distal than the mesial side (Table 3).

### 3.5. Risk of Bias and GRADE Analyses

**Figure 2:**
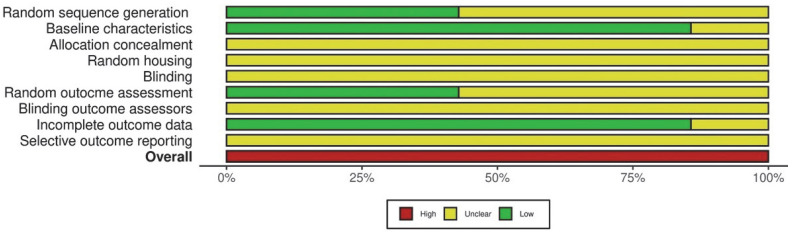
Risk of bias assessment using the SYRCLE tool.

All studies [12,15,16,17,20,22,23] had a high RoB (Figure 2) and a weak strength of recommendation (Table 4).

## 4. Discussion

Despite the well-recognized role of sclerostin in mechanotransduction and bone remodeling, a limited number of experimental studies have investigated its specific involvement in the context of OTM, yielding variable and, at times, contradictory findings [12,15,16,17,20,22,23]. To date, no systematic review has focused exclusively on assessing the effect of OTM on SE in the ABM. In this context, the authors of the present study conducted a systematic review of the available scientific evidence to evaluate patterns of SE during experimentally induced OTM [12,15,16,17,20,22,23]. A consensus emerging from the assessed studies [12,15,16,17,20,22,23] is that SE varies between compression and tension sites during OTM. Approximately 60% of these studies [12,15,16,23] reported an upregulation of SE on the compression side and a downregulation on the tension side, consistent with the biological mechanisms underlying bone remodeling. However, three studies [17,20,22] found no significant differences in SE between sites subjected to compressive and tensile forces, thereby introducing further complexity and highlighting the inconsistency within the currently existing evidence. During a meticulous evaluation of the methodologies employed in the included studies [12,15,16,17,20,22,23], the authors encountered notable inconsistencies in the methodology that impeded a coherent and standardized interpretation of the results. For instance, while some studies assessed SE using immunofluorescence [12,17,23], others [15,16,20] employed immunohistochemistry. The authors perceive that such variability in detection methods introduces potential inconsistencies in sensitivity and specificity, thereby complicating the direct comparison of results. Moreover, only one study [22] provided quantitative data regarding SE, which limited the ability to calculate effect-sizes or conduct a meta-analysis.

Prior SSE is a fundamental component of a well-designed study that ensures adequate statistical power to detect meaningful differences or associations, thereby minimizing the risk of type II errors [27,28]. In other words, without appropriate power calculations, the reliability of reported statistical significance remains questionable. Therefore, incorporating SSE is essential for ensuring the methodological rigor and reproducibility of both clinical and preclinical studies. It is worth noting that none of the studies included in the present systematic review [12,15,16,17,20,22,23] utilized power-adjusted data. On another note, the authors of the present systematic review observed an absence or inadequacy of control groups in some studies. For instance, Nam et al. [12] and Yiwen et al. [15] included untreated control groups with minimal or no SE in the alveolar bone, others [20,23] failed to report or differentiate expression in non-stressed tissues. Without an appropriate control group, distinguishing between force-induced alterations in SE and baseline physiological variations becomes challenging. Furthermore, inconsistencies in data collection further compromised the ability to synthesize findings across studies. For example, Nishiyama et al. [17] assessed SE at a single time point (day four), whereas Shu et al. [16] conducted evaluations at six different intervals over 21 days. Such differences limit the ability to assess publication bias (in the form of funnel plots) as well as the dynamic changes in SE and their correlation with biological phases of osseous remodeling during OTM. Consequently, the internal validity of findings seems weakened, and unaccounted variables may confound the conclusions regarding the mechanobiological role of sclerostin during OTM.

Assessment of the RoB is an essential step in evaluating the reliability of preclinical and clinical research findings [25]. It identifies systematic errors that may compromise the internal validity of a study and, consequently, the credibility of its outcomes. Likewise, the certainty of evidence (CoE) provides an evaluation of how confidently the available evidence can be applied in decision-making [26]. In other words, a high RoB and low CoE weaken the strength of recommendations, limit reproducibility, and constrain the translational value of experimental findings into clinical settings. Regrettably, all studies included in the present systematic review [12,15,16,17,20,22,23] had a high RoB and low CoE. Several methodological shortcomings seem to have contributed to this outcome. As mentioned above, none of the studies [12,15,16,17,20,22,23] were power adjusted, which raises the risk of type II errors. Moreover, randomization procedures, AC and blinding of investigators/outcome assessors were either not reported or insufficiently described in the studies assessed [12,15,16,17,20,22,23] thereby introducing selection and detection biases. Another factor that potentially contributed towards a high RoB and low CoE is the inconsistency in definition of control groups among the included studies [12,15,16,17,20,22,23]. Furthermore, variability in methods of assessing sclerostin expression (e.g., immunohistochemistry, immunofluorescence, or ELISA) limited comparability across studies and introduced potential measurement bias. Such methodological inconsistencies compromised the internal validity of the included studies [12,15,16,17,20,22,23] and the overall strength of the evidence base. It is suggested that future investigations require rigorous methodological refinement such as use of power calculations to ensure adequate sample sizes. Adherence to randomization protocols, AC, and blinding of investigators as well as outcome assessors may help minimize biases and strengthen internal validity of future studies. Moreover, use of standardized and well-defined control groups may facilitate differentiation between force-induced biological responses and baseline physiological variation. Furthermore, standardization of outcome measurement methods is also warranted; validated and reproducible techniques should be uniformly applied to enhance comparability across upcoming preclinical and/or clinical studies.

In addition to methodological variability, there are limitations in the translational applicability of the findings. Animal models, while offering controlled environments and eliminating systemic confounders such as smoking, diabetes, or hormonal fluctuations, may not accurately reflect the complexity of human clinical scenarios [5,29,30]. All animals in the reviewed studies were male, thereby excluding the potential modulatory effects of estrogen and other sex hormones on bone remodeling [31]. Estrogen, in particular, plays a crucial role in regulating bone metabolism by promoting osteoblastic activity and inhibiting osteoclastic resorption, thereby maintaining skeletal homeostasis [32]. Similarly, other hormones such as progesterone and androgens may exert synergistic or antagonistic effects on bone cell differentiation and activity [33]. Consequently, findings derived from exclusively male cohorts may not be generalizable to females, particularly given the well-documented sex-related differences in skeletal biology and periodontal bone response [32,33]. Future research should therefore incorporate both sexes to provide a more comprehensive understanding of the interplay between systemic hormones and bone remodeling in the context of OTM and periodontal health. It is worth mentioning that the mode of OTM in the included studies was restricted to mesial or distal displacement of a single molar, which may not accurately replicate clinical orthodontic scenarios where complex and multidirectional forces are often employed based on the severity and type of malocclusion. Therefore, it is challenging to determine the precise role of sclerostin in clinical scenarios due to the given limitations. Despite such methodological inconsistencies and limitations, emerging evidence suggests that sclerostin modulation may influence clinical orthodontics. It has been suggested that anti-sclerostin medications could be developed for use in jawbone-related conditions, as regulating alveolar bone turnover may offer a therapeutic approach for managing periodontal disease and facilitating orthodontic treatment [34]. Experimental results by Lu et al. [14] showed that local injections of sclerostin at the compression side of the alveolar bone accelerate OTM by promoting osteoclastogenesis. This suggests that sclerostin could be used to develop therapies that may selectively modulate bone remodeling processes. Such strategies could be particularly beneficial in cases requiring rapid OTM or in managing patients with poor compliance such as individuals undergoing clear aligner therapy. Moreover, the combination of both histological and molecular analyses may be more comprehensive in understanding the regulation of sclerostin in alveolar bone adaptation under orthodontic loading. It is highly recommended that prospective protocols with sufficient sample sizes ascertained by power calculations, standardized force application systems, and longitudinal designs for capturing temporal variations be conducted. Therefore, the authors of the present systematic review perceive that well-designed and power adjusted studies may open up new avenues for pharmacological interventions related to OTM with the potential of optimization of treatment effectiveness and restriction of side effects such as root resorption or excessive bone loss.

## 5. Conclusions

These findings suggest a potential regulatory role of sclerostin in ABM. However, the lack of standardized methodologies, absence of power calculations, inconsistent use of control groups, high RoB, low CoE, and heterogeneity in outcome measurement techniques substantially weaken the internal validity of individual studies and constrained the overall strength of recommendations. Further investigations based on power-adjusted data and standardized methodologies are needed to generate robust and reproducible evidence, clarify the precise mechanobiological role of sclerostin in ABM during OTM, and explore its potential translational applications in optimizing orthodontic treatment outcomes.

## Figures and Tables

**Table 1:** Characteristics of Animal Models.

Authors et al.	Species	Sample Size (n)	Sex	Age	Weight
Nam et al. [12]	Rats	NR	Male	7 weeks	200 g
Yiwen et al. [15]	Rats	24	Male	8 weeks	180–220 g
Shu et al. [16]	Rats	35	Male	6–8 weeks	180–220 g
Nishiyama et al. [17]	Mice	4	Male	8 weeks	NR
Ueda et al. [20]	Rats	NR	NR	NR	200–250 g
Yan et al. [22]	Rats	16	Male	10–12 weeks	NR
Odagaki et al. [23]	Mice	NR	Male	8 weeks	NR

NR = not reported.

**Table 2:** Study characteristics relating to orthodontic tooth movement.

Authors et al.	In-Group Tooth Movement (TM)	Orthodontic Force	Force Application Method	Euthanasia	Sclerostin Expression Analysis
Nam et al. [12]	Group 1: day one of OTM	40 g	Mesialization of maxillary first molar	NR	Immunofluorescence
	Group 2: day two of OTM				
	Group 3: day 6 of OTM				
	Control: sham-operated				
Yiwen et al. [15]	Group 1: OTM	50 g	Mesialization of maxillary first molar	0, 1, 3, 5, 7, 14 days	Immunohistochemistry
	Group 2: Untreated				
Shu et al. [16]	Group 1: 1 day of OTM	20 g	Mesialization of maxillary first molar	1, 3, 5, 7, 14, 21 days	Immunohistochemistry
	Group 2: 3 days of OTM				
	Group 3: 5 days of OTM				
	Group 4: 7 days of OTM				
	Group 5: 14 days of OTM				
	Group 6: 21 days of OTM				
Nishiyama et al. [17]	Group 1: OTM	10 g	Mesialization of maxillary first molar	4 days	Immunofluorescence
	Group 2: Untreated				
Ueda et al. [20]	Group 1: OTM	NR	Distalization of maxillary first molar	NR	Immunohistochemistry
	Group 2: Untreated				
Yan et al. [22]	Group 1: OTM	50 g	Mesialization of maxillary first molar	3 days	ELISA
	Group 2: Untreated				
Odagaki et al. [23]	Group 1: 0 days of OTM	10 g	Mesialization of maxillary first molar	0, 1, 5, 10 days	Immunofluorescence
	Group 2: 1 day of OTM				
	Group 3: 5 days of OTM				
	Group 4: 10 days of OTM				

OTM = orthodontic tooth movement, ELISA = enzyme-linked immunosorbent assay, NR = not reported.

**Table 3:** Sclerostin expression on compression and tension sites.

Authors et al.	Sclerostin Expression (Tension versus Compression Sites)		
	Control	Compression	Tension
Nam et al. [12]	Minimal expression ^^^	Increased ^^^	Increased ^^^
Yiwen et al. [15]	No sclerostin expression	Increased (day 5) ^^^	Increased (day 5) ^^^
Shu et al. [16]	No difference between mesial and distal sites ^^^	Increased ^^^	Decreased (day 1) ^^^
Nishiyama et al. [17]	Increased on the distal site compared to the mesial site ^^^	Similar to the control side ^^^	Decreased compared to the control side ^^^
Ueda et al. [20]	NR	Decreased ^†^	Decreased ^†^
Yan et al. [22]	14.18 pg/mL	22.85 pg/mL ^‡^	
Odagaki et al. [23]	NR	Increased (day 5) ^^^	Decreased (day 1) ^^^

NR: Not reported, ^^^ Sclerostin levels were not reported, ^†^ Reported from immunohistochemistry staining, ^‡^ Sclerostin expression was significantly higher on the OTM side than the control side (*p* < 0.01); however, precise values for SE on the tension and compression sites were not reported.

**Table 4:** GRADE Analysis.

Author et al.	Population	Study Design	Outcome Measures	Certainty of Evidence	Strength of Recommendation
Nam et al. [12]	Rats	Experimental	Immunofluorescence; In situ hybridization of SOST mRNA	Low	Weak
Yiwen et al. [15]	Rats	Experimental	IHC	Low	Weak
Shu et al. [16]	Rats	Experimental	IHC	Low	Weak
Nishiyama et al. [17]	Rats	Experimental	Immunofluorescence; Macroconfocal imaging	Low	Weak
Ueda et al. [20]	Rats	Experimental	IHC	Low	Weak
Yan et al. [22]	Rats	Experimental	ELISA	Low	Weak
Odagaki et al. [23]	Mice	Experimental	Immunofluorescence	Low	Weak

## Data Availability

Data is available on reasonable request.

## Supplementary Materials

The following supporting information can be downloaded at: https://www.mdpi.com/article/10.3390/dj13110513/s1, Table S1: Search strategy used for electronic databases.

## Abbreviations

The following abbreviations are used in this manuscript:

ABM	Alveolar Bone Matrix
CoE	Certainty of evidence
ELISA	Enzyme-linked Immunosorbent Assay
GRADE	Grading of Recommendations, Assessment, Development, and Evaluations
OTM	Orthodontic Tooth Movement
PICO	Population, Intervention, Comparison, Outcome
PRISMA	Preferred Reporting Items for Systematic Reviews and Meta-Analyses
PROSPERO	International Prospective Register of Systematic Reviews
RANKL	Nuclear Factor-κB Ligand
RoB	Risk of Bias
SE	Sclerostin Expression
SOST	Sclerostin Gene
SYRCLE	Systematic Review Centre for Laboratory animal Experimentation
