# Supplementary material for: Effect of Orthodontic Tooth Movement on Sclerostin Expression in Alveolar Bone Matrix: A Systematic Review of Studies on Animal Models
Source: Dent J (Basel). 2025 Nov 4;13(11):513. doi: 10.3390/dj13110513 (PMC12651180; doi:10.3390/dj13110513)
Supplement: Supplementary file 1 [file dentistry-13-00513-s001.zip › dentistry-3843436-supplementary.pdf]

Supplemental Table S1. Search strategy used for electronic databases

Database	Results	Keywords – Mesh terms
PubMed	37	("Sost protein, rat" [Supplementary Concept] OR "Sost protein, mouse" [Supplementary Concept] OR Sclerostin OR SOST) AND ("Tooth Movement Techniques"[Mesh] OR "Orthodontic Tooth Movement" OR "Tooth Movement")
Web of Science	56	("Sost protein, rat" OR "Sost protein, mouse" OR Sclerostin OR SOST) AND ("Tooth Movement Techniques" OR "Orthodontic Tooth Movement" OR "Tooth Movement")
Embase	134	('Sost protein, rat':tn OR 'Sost protein, mouse':tn OR Sclerostin OR SOST) AND ('Tooth Movement Techniques'/exp OR 'Orthodontic Tooth Movement' OR 'Tooth Movement')
